# Identification of a novel ovine LH-beta promoter region, which dramatically enhances its promoter activity

**DOI:** 10.1186/s40064-015-1182-5

**Published:** 2015-09-01

**Authors:** Redouane Aherrahrou, Zouhair Aherrahrou, Jeanette Erdmann, Mohieddine Moumni

**Affiliations:** Institute for Integrative and Experimental Genomics (IIEG), Luebeck University, Luebeck, Maria-Goeppert-Str. 1, 23562 Lübeck, Germany; Department of Biology, Faculty of Sciences, Moulay Ismail University, Zitoune, BP 11201, 50000 Meknes, Morocco

**Keywords:** Reproduction, LH-beta, Promoter, Luciferase assay

## Abstract

The luteinizing hormone beta subunit (*LH*-*beta*) gene plays a critical role in reproduction. In order to characterize and analyze the promoter region of LH-beta in sheep, a genomic library was constructed in phage lambda *gt* 10 and screened. A novel region of 1,224 bp upstream from the targeted LH-beta gene was identified. Blasting this sequence showed a perfect homology for the first 721 bp sequence with an upstream ovine LH-beta sequence in the database. However, the remaining 5′-503 bp showed no sequence matching. DNA from Moroccan breeds was isolated and the whole region was amplified and confirmed by sequencing. To further confirm the promoter activity of this region, an in vitro analysis using a luciferase assay was carried out. An increase in the promoter activity of the whole region was demonstrated compared to the empty vector. More interestingly, the unpublished region significantly enhanced the promoter activity compared to the known region alone. To predict putative transcription factor binding-sites (TFBSs), an in silico analysis was performed using the TFSEARCH program. The region features many TFBSs and contains two palindrome sequences of 17- and 18-bp. Taken together, a novel region was identified and confirmed in sheep which contained a promoter activity rich with binding sites for a putative regulatory element as shown in silico.

**Electronic supplementary material:**

The online version of this article (doi:10.1186/s40064-015-1182-5) contains supplementary material, which is available to authorized users.

## Background

Sheep breeding in Morocco plays an important role in several areas of society, such as in traditional life and in the economy, and it also represents the principle source for red meat. Luteinizing hormone (LH) and follicle-stimulating hormone (FSH) are considered as the main hormones which directly regulate reproductive performance (Campbell et al. 1999; Mullen et al. 2013). Genetic factors were known to affect directly or indirectly the level of theses hormones. In Booroola, a *FecB* locus was recently identified as controlling breeding performance in this breed and evidence demonstrated the effect of a *FecB* genotype within a specific neonatal period on the plasma levels of FSH and LH in ewe and ram lambs of this breed (McNatty et al. 1994; Isaacs et al. 1995; Heath et al. 1996). Here we hypothesized that these hormones could be considered as important downstream targets for several upstream genetic variants in sheep, especially those located in their own promoter regions. The hormones belong to the glycoprotein heterodimers family, which consist of a common alpha subunit and a unique beta subunit that gives each hormone its biological and immunological specificity, and encoded by a single-copy gene (Pierce and Parsons 1981).

In this study we focused on lutropin hormone, and more particularly the beta subunit (LH-beta) which is secreted by the anterior pituitary (Sairam and Li 1975; Counis et al. 1993; Moumni et al. 1994; Lerrant et al. 1995; Pelletier et al. 1995). This hormone plays a determinant role in reproduction where it stimulates gonadal steroidogenesis and gametogenesis (Ma et al. 2004). Furthermore, it has been shown that the *LH*-*beta* subunit gene reveals changes and polymorphisms even within species (Maston and Ruvolo 2002; Alevizaki and Huhtaniemi 2002). As such, the characterization of the LH-beta promoter region in sheep is of crucial importance.

In this paper we aimed to identify and characterize the promoter region of LH-beta.

## Results

### Identification of a novel promoter sequence region in the *LH*-*beta* gene

**Fig. 1:**
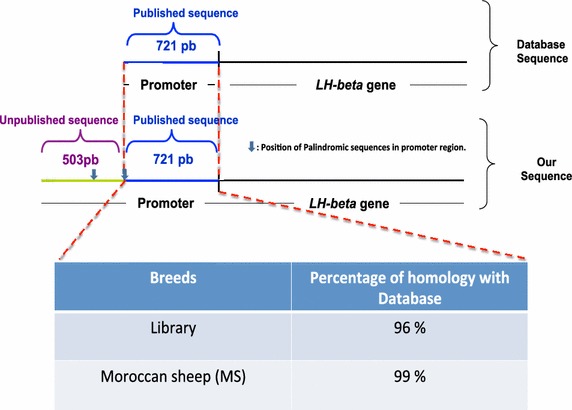
Schematic representation of the promoter region of the *LH beta* gene from a library screening of the Moroccan sheep database sequence: Homology sequence analysis revealed that the two sequences shared more than 96% homology with the reference sequence available in the database after alignment (Brown et al. 1993).

The *LH*-*beta* gene was cloned from a sheep genomic library, which was constructed in phage lambda *gt* 10 and screened. Six clones were selected and the corresponding DNA products were sequenced and analyzed using a Blast program. The analysis of the identified clone showed a perfect homology with the sequence of ovine LH-beta published in the database (http://blast.ncbi.nlm.nih.gov, Brown et al. 1993). However, our identified sequence contained an additional promoter fragment of 503 bp (in the 5′ side), which has not yet been published (Fig. 1 and Additional file 1: Figure S1 in the supporting information).

### Enhancement of the promoter activity by the novel identified region

**Fig. 2:**
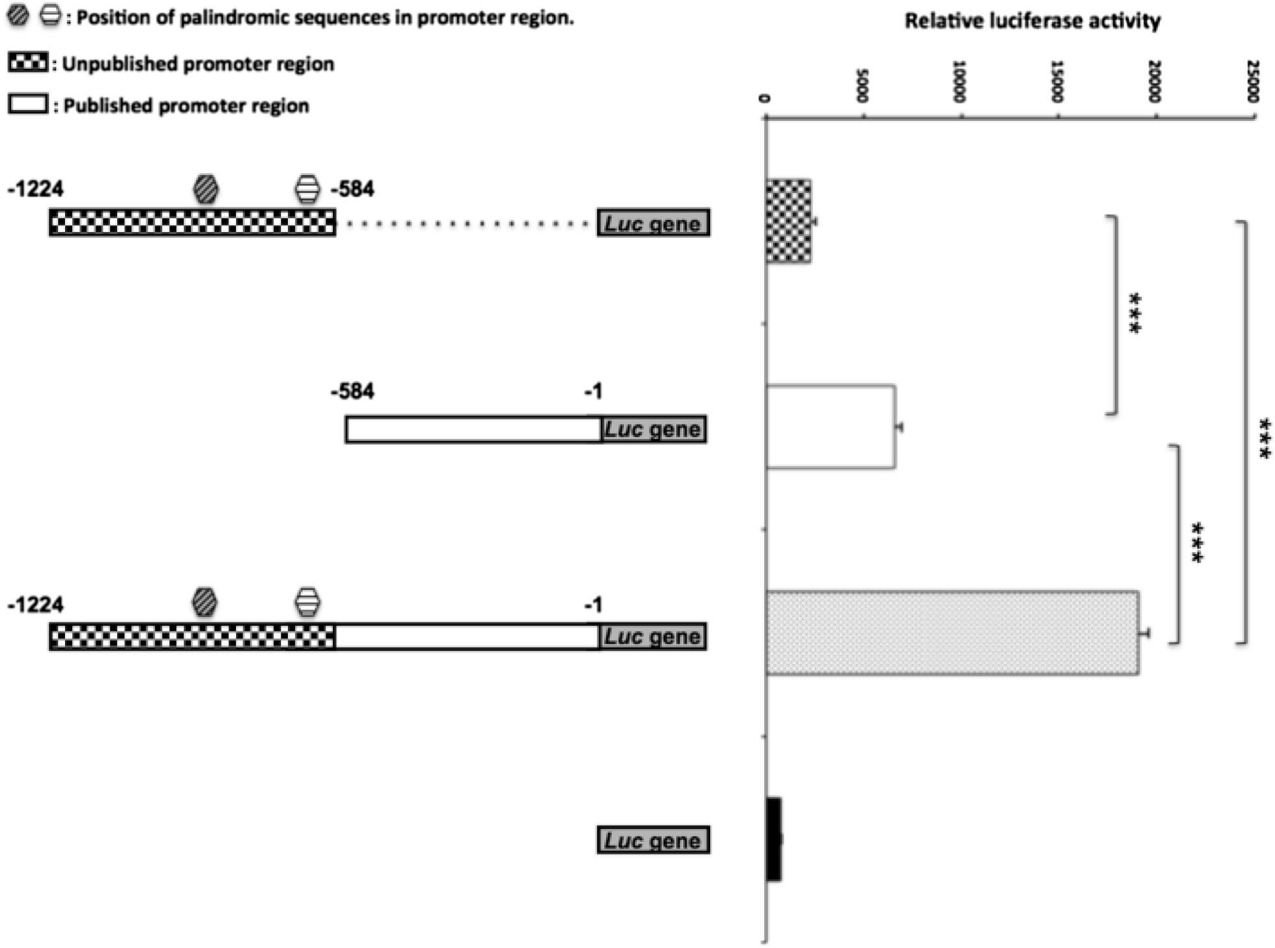
Investigation of LH-beta promoter region activity in Moroccan sheep. The promoter region including the published, unpublished and entire regions were amplified and analyzed for promoter activity using the luciferase assay. The luciferase gene alone was used as a negative control. The position of the two palindromic sequences is also shown. P-values of <0.05 were considered as statistically significant (*p < 0.05, **p < 0.01, and ***p < 0.001).

In order to test whether the unpublished novel promoter region is relevant for the promoter activity, fragments including either published, unpublished or the whole promoter region were amplified on genomic DNA from Moroccan sheep and analyzed using luciferase assays. As expected, the published region alone showed a higher promoter activity when compared to the empty vector. The novel unpublished region alone demonstrated a moderate increase in promoter activity compared to the empty vector. However, when both fragments (published and unpublished) were combined a 65% enhancement of the promoter activity was shown compared to that seen with the formerly published promoter region (Fig. 2).

### Prediction of relevant regulatory elements within the promoter region using in silico bioinformatics tools

**Fig. 3:**
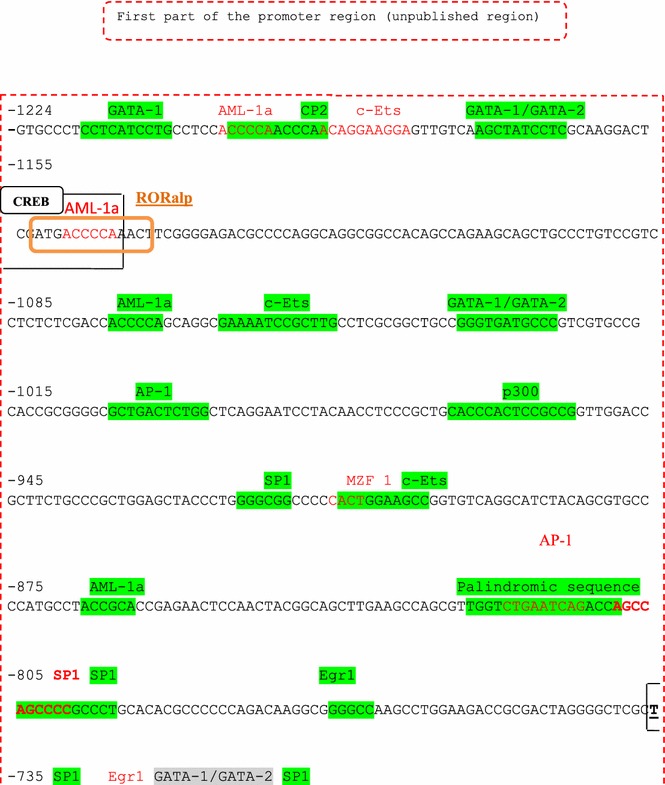
In-silico transcription factor binding sites (TFBS) analysis of the whole promoter region of the *LH beta* gene. The in silico analysis of the promoter region was carried out using the TFSEARCH software program. The localization of each TF is shown in the figure according to its position from the ATG start codon. The first (unpublished) part of the promoter is shown.

**Fig. 4:**
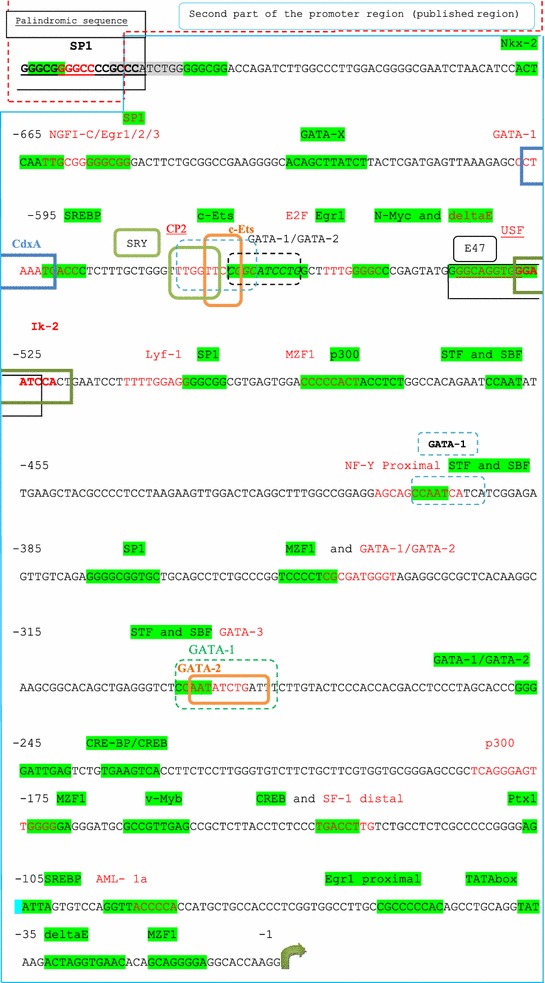
In-silico transcription factor binding sites (TFBS) analysis of the whole promoter region of the *LH beta* gene. The in silico analysis of the promoter region was carried out using the TFSEARCH software program. The localization of each TF is shown in the figure according to its position from the ATG start codon. The second (published) part of the promoter is shown.

The TFSEARCH in silico program was used to predict potential regulatory elements within the whole region containing previously and newly identified regions. The analysis of the whole region revealed more than 30 putative regulatory elements (Figs. 3, 4). In addition, two extended perfect palindrome sequences of 17- and 18-bp were also identified. Palindromic sequences are thought to be regulatory sites for steroid hormones (Aumais et al. 1996).

## Discussion

The *LH*-*beta* gene encodes for a key glycoprotein hormone which is expressed in the pituitary gland and which regulates steroid synthesis in the testes and ovaries. As such it plays a decisive role in the development and maturation of sexual organs (Tremblay and Drouin 1999; Walsh and Shupnik 2009).

Genetic factors affecting the level of these hormones are functionally relevant and may explain variations in reproductive performance seen across many mammalian species. To understand the role of LH-beta hormone levels in reproduction, previous studies have tried to increase or decrease the level of this hormone in various mammalian species. Mice deficient in LH-beta were found to be infertile in both sexes (Ma et al. 2004; Kumar 2005). The LH-beta knockout males demonstrated reduced testes size (by 75% compared to controls). The LH-beta knockout females also demonstrated a hypogonadal phenotype where ovarian histology reveals abnormal antral and preovulatory follicles as well as the absence of corpora lutea (Ma et al. 2004; Kumar 2005). This mouse model represents an extreme case where the LH-beta is completely absent. However, moderate forms of hypogonadism can be also driven by single nucleotide polymorphism (SNP), especially in regulatory elements affecting regions that subsequently impact on the level of LH-beta either directly or indirectly. For example the Booroola breed is known for its high prolificacy worldwide (Davis et al. 1982; Montgomery et al. 2001; Souza and Baird 2004; Mishra et al. 2009; Ruoss et al. 2009; Reader et al. 2012).

A locus on chromosome 6 (*FecB*) has been identified to control the litter size of prolific breeds such as Booroola Merino, Garole and Javanese and to affect the plasma levels of FSH and LH within a specific neonatal period (McNatty et al. 1994; Isaacs et al. 1995; Heath et al. 1996; Wilson et al. 2001; Souza et al. 2001; Mulsant et al. 2001). Human mutations have been reported amongst three infertile men who showed an absence of FSH (Phillip et al. 1998; Layman et al. 2002; Lindstedt et al. 2005). Further functional studies demonstrated only 50% of the promoter activity of FSHB in the T allele carriers compared to the wild-type G allele carriers (Hoogendoorn et al. 2003; Grigorova et al. 2008). As such, the level of FSH and LH is crucial in reproduction, and screening for novel sequences especially in the promoter regions for both hormones is of major importance.

In this study we focused on the luteinizing hormone beta subunit (*LH*-*beta*) where our intention was to characterize the promoter region of the LH-beta gene. After screening of a genomic library a novel additional upstream promoter region was identified. This sequence was used as a template to design primers and amplify the corresponding entire promoter region on DNA isolated from Moroccan sheep. The amplified sequence shares 96% homology with the sequence from the screened library and 99% homology with the sequence published in the database. This difference may be explained by the high level of heterogeneity seen amongst sheep breeds worldwide. High intra-species genetic variability has indeed been reported between Tunisian (Khaldi et al. 2010), Egyptian (Mahfouz et al. 2008) Brazilian (Paiva et al. 2005) and Maltese breeds (Blundell and Felice 2006).

To test whether the unpublished upstream 503 bp promoter region displays promoter activity, we used the in vitro luciferase assay for this region or combined with the published region. Interestingly, the whole promoter region including the novel unpublished region demonstrated a much higher increase in promoter activity compared to the published region alone. This sequence can certainly be used in the future to test for SNPs within the LH-beta promoter that might regulate LH-beta transcription in cis and which may therefore explain variations in reproduction performance amongst different sheep breeds.

In order to understand at a molecular level whether this region includes relevant transcription factors (TF), we performed in silico analysis of the whole promoter region of LH-beta to predict regulatory elements using the TFSEARCH program. Interestingly, more than 30 regulatory elements were identified as well as two previously described perfect palindromes of 17 and 18 bp length. All these in silico identified regulatory elements require further in vitro or in vivo confirmation. Palindromic sequences are known to play a key role in sites regulated by steroid hormones (Aumais et al. 1996). The re-identification of the two palindromic sequences by the software program we used confirms the effectiveness of the program. Moreover, additional potential TF binding sites were also found in the unpublished region to play a role in reproduction, a fact, which supports our hypothesis regarding the decisive role of LH-beta in reproduction. Amongst these the transcription factors GATA-1, GATA-2, AML1a, P300, CBP, Egr-1 and SP1 have all been characterized (LaVoie 2003; Viger et al. 2004). As such genetic variations within these regulatory element sites could conceivably influence the transcription of LH-beta transcription und in turn the levels of secreted hormone.

## Conclusion

In this work we screened and identified a novel promoter region for LH-beta. We confirmed and functionally tested the relevance of this novel region using the in vitro luciferase assay. Using in silico analysis, novel regulatory elements that may play a role in the regulation of LH-beta were predicted, and these are also in need of further functional analysis.

## Methods

### Ethical guidelines

Animal experiments were carried out according to the guidelines stated in the Guide for the Care and Use of Agricultural Animals in Research and Teaching of Meknès-Tafilalet, Morocco.

### Screening of the sheep genomic library

A genomic library was constructed with sheep DNA and cloned into phage lambda gt10 using a *Bam*HI restriction enzyme site. Approximately six million clones were then plated onto agar media transfecting *E. coli* C600 and C600-Hfl. The first strain provides an estimate of the total number of phages obtained, the second allows us to estimate the amount of recombinants. The library was then screened with the ovine LH-beta cDNA labeled with 32P using the random priming method (d’Angelo-Bernard et al. 1990). Six positive clones were then identified and isolated. They underwent several purification steps and were then sub-cloned into the vector pUC 19 and sequenced using Sanger method.

### DNA isolation

For DNA isolation, blood samples were collected from a Moroccan sheep and incubated in lysis buffer containing NaCl 75 mM, EDTA 80 mM (pH = 8), SDS 0.5%, Tris–HCl (pH = 8) and 200 μg/ml of Proteinase K at 50°C with shaking for 3 h until complete digestion. The genomic DNA was gently extracted twice; with an equal volume of phenol/chloroform (3:1) V/V (chloroform saturated with isoamylic alcohol 42:1 V/V). A centrifugation for 15 min at 2,800×*g* was then performed after which the DNA in the aqueous phase was precipitated using 1/10 of 3 M NaAc (pH = 6) and 0.8 volumes of isopropanol. The DNA was then washed twice with 70% ethanol at room temperature and left to dry. The DNA was then resuspended in TE (Tris–EDTA) buffer and stored at −20°C. DNA concentration and purity were determined using a UV spectrophotometer at 260 and 280 nm.

### PCR amplification

Fragments containing the unpublished region (503 bp), the published region (721 bp) or both together (1,224 bp) from the LH-beta promoter sheep were amplified using PCR and the primer pairs were designed from the sequence of the ovine *LH beta* gene obtained through the screening of the genomic library (Table 1). *kpn*I and *Sac*I restrictions enzyme sites were added to the 5′ end of the primers. PCR products were obtained from the amplified genomic DNA of different samples using the 5′PRIME PCR Mastermix kit (VWR International GmbH, Germany). The products were separated by electrophoresis in a low melting-point agarose gel and visualized using SYBR-Green I (Invitrogen). The expected PCR products were then cut from the gel and purified using the NucleoSpin^®^ Extract II kit (Macherey-Nagel).

**Table 1:** List of primers used for generating the different lengths of the promoter regions

Forward LH-*Kpn*I	Reverse LH-*Sac*I
5′-ggtaccTTTGCTGGGTTTGGTTCC-3′	5′-gagctcCCTTGGTGCCTCCCCTGCTGTGTTC-3′
5′-ggtaccTGTGCCCTCCTCATCCTG-3′	5′-gagctcGAGTTAAAGAGCCTAAATCACCCTC-3′
5′-ggtaccTGTGCCCTCCTCATCCTG-3′	5′-gagctcCCTTGGTGCCTCCCCTGCTGTGTTC-3′

### Cloning into the TOPO vector

PCR fragments were cloned into the pCR^®^2.1-TOPO^®^ vector using the TOPO TA Cloning Kit according to the manufacturer’s instructions (Invitrogen). Plasmids were then isolated using the Miniprep Kit as described by the manufacturer (QIAprep Spin Miniprep Kit, Qiagen) and stored at −20°C until sequencing.

### Sequencing

Sequencing of PCR products was performed on both strands using a commercial sequencing service (Seqlab, Goettingen, Germany).

### Promoter sequence analysis

The alignment program (blast program) was used for DNA sequence comparison. TFSEARCH: Searching Transcription Factor Binding Sites (v.1.3; Yutaka Akiyama, Kyoto University Real World Computing Partnership, Japan) was used to search for transcription binding sites.

### Plasmid constructs

The three cloned LH-beta promoter fragments were cut from the pCR^®^2.1-TOPO^®^ vector (Invitrogen) using *kpn*I and *Sac*I restriction enzymes and inserted into the pGL4.10 reporter plasmid (Promega). All sequences were verified both by restriction digestion analysis and direct sequencing. Sequencing was performed on both strands using a commercial sequencing service (Seqlab, Goettingen, Germany).

### Cell culture

For the expression of ovine *LH*-*beta* promoter gene, a non-endocrine cell line, Human Embryonic Kidney cells (HEK293), was used (Jiang et al. 1999; Lamminen et al. 2002). HEK293 were cultured in high glucose Dulbecco’s modified Eagle’s medium GLUTAMAX, pyruvate (Invitrogen) supplemented with 10% heat-inactivated fetal bovine serum (PAA) and 100 U penicillin/streptomycin (Biochrom) in a humidified 5% CO_2_ incubator at 37°C.

### Cell transfection

The constructs were transfected into HEK293 cells using TransFectin reagent (BIO-RAD). HEK293 cells were plated into 24-well tissue culture plates (Nunc) at 2 × 10^5^ cells per well in 1 ml of the appropriate complete growth medium 18–24 h prior to transfection. Cells were transfected using TransFectin reagent at 60–80% confluency in antibiotic-free medium according to the manufacturer’s instructions. We used 2 µg of constructs and 30 ng of the pGL4.74 vector that expresses Renilla luciferase under the control of a HSV-TK promoter as an internal control to normalize variations in transfection efficiency. After 24 h the media was changed and the cells were incubated further for 48 h. TransFectin reagent was used for each transfection reaction.

### Luciferase assay

After 48 h of transfection HEK293 cells were washed twice with PBS (without Ca^2+^ and Mg^2+^) and harvested using 100 µl of l× Passive Lysis Buffer (Promega). Cell extracts were transferred into a 1.5 ml microcentrifuge tube and clarified by centrifugation for 1 min at 13,000×*g* and room temperature. Complete cell lysis was achieved using one freeze–thaw cycle that consisted of incubation at −80°C followed by a rapid thawing at 37°C. 25 µl of the supernatants from each sample were transferred to a white 96 well plate (Costar). Activity of firefly and Renilla luciferase was measured after 24–48 h incubation using the Dual Luciferase Reporter Assay System (Promega) according to the manufacturer’s instructions. All the transfection experiments were carried out in triplicate, and in at least three independent experiments.

### Statistical analysis

In order to control for transfection efficiency and cell recovery, the luciferase activity was normalized according to the manufacturer’s protocol. All the experiments were performed three times independently (at least), and each of which measured in triplicates. Data are presented as mean ± standard error of the mean (SEM). For statistical analyses we used the unpaired t-test (GraphPad Prism, Version 6.0 d). P-values of <0.05 were considered as statistically significant (*p < 0.05, **p < 0.01 and ***p < 0.001).

## Additional file

Additional file 1. Figure S1. Alignment of the promoter region sequence from the library screening with the database sequence of the Moroccan sheep breeds: The promoter region was amplified and sequenced. The sequences were then aligned and the genetic variations between the three sequences were marked for mutations, insertion and deletion in gray. MS: Moroccan Sheep, library: sheep genomic library sequence constructed in phage lambda *gt* 10. Reference: Sequence from the database (Brown et al. 1993).
