# Supplementary material for: Identification of a novel ovine LH-beta promoter region, which dramatically enhances its promoter activity
Source: Springerplus. 2015 Sep 1;4:466. doi: 10.1186/s40064-015-1182-5 (PMC4554545; doi:10.1186/s40064-015-1182-5)
Supplement: Additional file 1. Figure S1. — Alignment of the promoter region sequence from the library screening with the database sequence of the Moroccan sheep breeds: The promoter region was amplified and sequenced. The sequences were then aligned and the genetic variations between the three sequences were marked for mutations, insertion and deletion in gray. MS: Moroccan Sheep, library: sheep genomic library sequence constructed in phage lambda gt 10. Reference: Sequence from the database (Brown et al. 1993). [file 40064_2015_1182_MOESM1_ESM.docx]

**SUPPORTING INFORMATION**

**Figure.S1: Alignment of the promoter region sequence from the library screening with the database sequence of the Moroccan sheep breeds:** The promoter region was amplified and sequenced. The sequences were then aligned and the genetic variations between the three sequences were marked for mutations, insertion and deletion in gray. MS: Moroccan Sheep, library: sheep genomic library sequence constructed in phage lambda *gt* 10. Reference: Sequence from the database (Brown et al., 1993).

**Figure.S1**

**First part of the promoter region (unpublished region: UP)**

-1224

Reference ------------------------------------------------------------

Library TGTGCCCTCCTCATCCTGCCTCCACCCCAACCCAACAGGAAGGAGTTGTCAAGCTATCCT

MS TGTGCCCTCCTCATCCTGCCTCCACCCCAACCCAACAGGAAGGAGTTGTCAAGCTATCCT

************************************************************

Reference ------------------------------------------------------------

Library CGCAAGGACTCGATGACCTCAAACTTCGGGGAGACGCCCCAGGCAGGCGGCCACAGCCAG

MS CGCAAGGACTCGATGACCCCAAACTTCGGGGAGACGCCCCAGGCAGGCGGCCACAGCCAG

****************** *****************************************

Reference ------------------------------------------------------------

Library AAGCAGCTGCCCTGTCCGTCCTCCTCTCGACCACCCCAGCAGGCGAAAATCCGCTCGTCG

MS AAGCAGCTGCCCTGTCCGTCCTCCTCTCGACCACCCCAGCAGGCGAAAATCCGCTTGCCT

******************************************************* * *

Reference ------------------------------------------------------------

Library C--GGCTGCCGGGTGATGCCCGTCG-TGCGCACCGCGGGGCGCTGACTCTGGCTCAGGAA

MS CGCGGCTGCCGGGTGATGCCCGTCGTGCCGCACCGCGGGGCGCTGACTCTGGCTCAGGAA

* ********************** ********************************

Reference ------------------------------------------------------------

Library TCCTACAACCTCCCGCTGCACCCACTCCGCCGGCTGGACCGCTTCTCGCGGCTGGAGCTG

MS TCCTACAACCTCCCGCTGCACCCACTCCGCCGGTTGGACCGCTTCTGCCCGCTGGAGCTA

********************************* ************ * *********

Reference ------------------------------------------------------------

Library CCCTGG-GCGGCCCCCACTGGAAGCCGGTGTCAGGCATCTACAGCGTGCCTCATGCCTAC

MS CCCTGGGGCGGCCCCCACTGGAAGCCGGTGTCAGGCATCTACAGCGTGCCTCATGCCTAC

****** *****************************************************

Reference ------------------------------------------------------------

Library CGCACCGAGAACTCCAACTACGGCAGCTTGAAGCCAGCGTTGGTCTGAATCAGACCAGCC

MS CGCACCGAGAACTCCAACTACGGCAGCTTGAAGCCAGCGTTGGTCTGAATCAGACCAGCC

************************************************************

Reference ------------------------------------------------------------

Library AGCCCCGCCCTGCACACGCCCCCCAGACAAGGCGGGGCCAAGCCTGGAAGACCGCGACTA

MS AGCCCCGCCCTGCACACGCCCCCCAGACAAGGCGGGGCCAAGCCTGGAAGACCGCGACTA

************************************************************

**Second part of the promoter region (published region: P)**

-721 Reference ----------------------CCCCATCTGGGGGCGGACCAGATCTTGGCCCT-TGGAC

Library GGGGCTCGCTGCGGGCACCGGCCGCAATCTGGGGGCGGACCAGATCTTGGCCT---GGAC

MS GGGGCTCGCTGGGCGGGGCCCCGCCCATCTGGGGGCGGACCAGATCTTGGCCCTTGGACG

* ************************** *

Reference GGGCGAATCTAACATCCACTCAATTGCGGGGGCGGGACTTCTGCGGCCGA-AGGGCACAG

Library GGGCGAATCTAACATCCACTCAATTGCGGGGGCGGGACTTCTGCGGCCGAAGGGGCACAG

MS GGGCGAATCTAACATCCACTCAATTGCGGGGGCGGGACTTCTGCGGCCGAAGGGGCACAG

************************************************** ********

Reference CTTATCTTACTCGATGAGTTAAAGAGCCTAAATCACCCTCTTTGCTGGGTTTGGTTCCGG

Library CTTATCTTACTCGATGAGTTAAAGAGCCTAAATCACCCTCTTTGGTGGGTTTGGTTCCGG

MS CTTATCTTACTCGATGAGTTAAAGAGCCTAAATCACCCTCTTTGCTGGGTTTGGTTCCGG

******************************************** ***************

Reference GATCCTGGCTTTTGGGGCCCCGAGTATGGGGCAGGTGGGAATCCACTGAATCCTTTTTGG

Library CATCCTGGCTTTTGGGG-CCCGAGTATGGGGCAGGTGGGAATCCACTGAATCCTTTTTGG

MS CATCCTGGCTTTTGGGG-CCCGAGTATGGGGCAGGTGGGAATCCACTGAATCCTTTTTGG

**************** ******************************************

Reference AGGGGCGGCGTGAG----------CTACCTCTGGCCACAGAATCCAATATTGAAGCTACG

Library AGGGGCGGCGTGAGTGGACCCCCACTACCTCTGGCCACAGAATCCAATATTGAACGTACG

MS AGGGGCGGCGTGAGTGGACCCCCACTACCTCTGGCCACAGAATCCAATATTGAAGCTACG

************** ****************************** ****

Reference CCCCTCCTAAGAAGG-----------CTTTGGCCGGAGAGCAGCCAATCATCATCGGAGA

Library CCCCTCCTAAGAAGATGGACTCAGCT--TGGCCGGAGGAGCAGCCAATCATCATCGGAGA

MS CCCCTCCTAAGAAGTTGGACTCAGGCTTTGGCCGGAGGAGCAGCCAATCATCATCGGAGA

************** * * * * ***********************

Reference GTTGTCAGAGGGGCGGTGCTGCAGCCTCTGCCCGGTCCCCTCGCGATGGGTAGAGGCGCG

Library GTTGTCAGAGGGCGGTGCTGCAGCCT--TCTGCCGGTCCCTCGCGATGGGTAGAGGCGCG

MS GTTGTCAGAGGGGCGGTGCTGCAGCCTCTGCCCGGTCCCCTCGCGATGGGTAGAGGCGCG

************ * * * * * ***********************

Reference CTCACAAGGCAAGCGGCACAGCTGAGGGTCTCCAATATCTGATTTCTTGTACTCCCACCA

Library CTCACAAGGCAAGCGGCACAGCTGAGGGTCTCCAATATCTGATTTCTTGTACTCCCACCA

MS CTCACAAGGCAAGCGGCACAGCTGAGGGTCTCCAATATCTGATTTCTTGTACTCCCACCA

************************************************************

Reference CGACCTCCCTAGCACCCGGGGATTGAGTCTGTGAAGTCACCTTCTCCTTGGGTGTCTTCT

Library CGACCTCCCTAGCACCCGGG-ATTGAGTCTGTGAAGTCACCTTCTCCTGG-GTGTCTTCT

MS CGACCTCCCTAGCACCCGGGGATTGAGTCTGTGAAGTCACCTTCTCCTTGGGTGTCTTCT

******************** *************************** * *********

Reference GCTTCGTGGTGCGGGAGCCGCTCAGGGAGTTGGGGGAGGGATGCGCCGTTGAGCCGCTCT

Library GCTTCGT-GGTCGGGAGCCGCTCAGGGAGTTGGGGGAGGGATCGCCCGTTGAGCCGCTCT

MS GCTTCGTGGTGCGGGAGCCGCTCAGGGAGTTGGGGGAGGGATGCGCCGTTGAGCCGCTCT

******* * ******************************* ***************

Reference TGCCTCTCCCTGACCTTGTCTGCCTCTCGCCCCCGGGGAGATTAGTGTCCAGGTTACCCC

Library TGCCTCTCCCTGACCTTGTCTGCCTCTCGCCCCGGGAGTTAGTGTCCA----GTTACCCC

MS TACCTCTCCCTGACCTTGTCTGCCTCTCGCCCCCGGGGAGATTAGTGTCCAGGTTACCCC

* ******************************* ** * * * ********

Reference ACCATGCTGCACC---CTCGGCGCTTGCCGCCCCCACAGCCTGCAGGTATAAGACTAGGT

Library AC-CATGCTCAGCCCTCGGTGGCCTTGCCGCCCCCACAGCCTGCAGGTATAAGCT-AGGT

MS ACCATGCTGCCACCCTCGGTGGCCTTGCCGCCCCCACAGCCTGCAGGTATAAGACTAGGT

** * * * * ****************************** ****

-1

Reference GAACACAGCAGGGGAGGCACCAAGG

Library GAACACAGCAGGGGAGGCACCAAGG

MS GAACACAGCAGGGGAGGCACCAAGG

*************************
